# Epidemiology and Molecular Characterization of Feline Calicivirus in Beijing, China

**DOI:** 10.3390/ani15040494

**Published:** 2025-02-10

**Authors:** Daoqi Wang, Jingru Zhu, Hanyu Yang, Yanli Lyu

**Affiliations:** 1 College of Veterinary Medicine, China Agricultural University, Beijing 100193, China; dodgewdq@163.com (D.W.); jingru_zhu@163.com (J.Z.); yanghanyu0208@163.com (H.Y.); 2 China Agricultural University Veterinary Teaching Hospital, Beijing 100193, China

**Keywords:** feline calicivirus, risk factors, phylogenetic analysis, VP1, recombination

## Abstract

Feline calicivirus (FCV) is an infectious pathogen in cats that mainly causes upper respiratory tract disease. Enhancing our understanding of the epidemiological characteristics of FCV can contribute to better strategies against FCV infection. To investigate the prevalence of FCV in Beijing, explore the risk factors associated with FCV infection and elucidate its genetic evolutionary characteristics. Cats (*n* = 402) from the China Agricultural University Veterinary Teaching Hospital (CAUVTH) were investigated from June to December in 2023. The rate of FCV-positive cats in the sample examined was 31.3%. Risk factors significantly associated with FCV infection were age, vaccination status and residential density by Logistic regression analysis. Phylogenetic analysis of completed genomes revealed a radial phylogeny, with no obvious geographical clustering. Amino acid analysis at different sites of E region of the major capsid protein revealed variable neutralizing antibody epitopes, while feline junctional adhesion molecule-A (fJAM-A) binding sites remained conserved. Additionally, the first FCV recombinant isolate was detected in Beijing, originating from two 2019 isolates collected in the city. This study elucidates the molecular epidemiology and genetic diversity of FCV in Beijing, which provides valuable insights for the development of effective measures for FCV prevention and control.

## Simple Summary

Feline calicivirus (FCV) commonly affects cats, causing upper respiratory disease. This study investigated the prevalence of FCV in Beijing, explored the risk factors associated with FCV infection and elucidated its genetic evolutionary characteristics. A total of 402 cats from the China Agricultural University Veterinary Teaching Hospital (CAUVTH) were investigated from June to December in 2023. The rate of FCV-positive cats in the sample examined was 31.3%. Risk factors significantly associated with FCV infection were age, vaccination status and residential density. A phylogenetic analysis of completed genomes revealed a radial phylogeny, with no obvious geographical clustering. An amino acid analysis of the E region of the major capsid protein revealed variable neutralizing antibody epitopes, while feline junctional adhesion molecule-A (fJAM-A) binding sites remained conserved. Additionally, we identified the first recombinant strain of FCV in Beijing, created from two earlier strains in 2019. This study elucidates the molecular epidemiology and genetic diversity of FCV in Beijing, helping us improve prevention and control strategies to protect cats from this disease.

## 1. Introduction

Feline calicivirus (FCV) is commonly found in cat populations. The virus was first isolated in 1957 [1] and is now widespread in the United States, Europe and parts of Asia [2,3,4]. Typical clinical symptoms caused by feline calicivirus infection include upper respiratory tract disease (URTD), fever, mouth ulcers, chronic gingivostomatitis, pneumonia, enteritis, paw and mouth disease and lameness [5,6,7,8]. In recent years, highly virulent strains of calicivirus have emerged globally, causing virulent systemic disease (VSD) including systemic inflammatory responses, disseminated intravascular coagulation (DIC), multiple organ failure and mortality rates ranging from 30% to 70% [9,10], in addition to the previously mentioned symptoms. Both infected and asymptomatic carrier cats can continuously shed the virus [11], facilitating the persistent spread of FCV among feline populations.

FCV is a non-enveloped, single-stranded, positive-sense RNA virus classified under the genus *Vesivirus* within the family *Caliciviridae* [12]. The genome of FCV is about 7.7 kb and encodes three open reading frames (ORFs) [13]. ORF1 (approximately 20–5311 nucleotides) encodes six non-structural proteins. Among them, Pro-Pol suppresses host gene expression [14], and P39 cleaves host nucleotides [15]. ORF2 (approximately 5314–7320 nucleotides) encodes the major capsid protein, VP1. The amino acid sequence of VP1 is divided into six regions, labeled A–F. Region E is further subdivided into two hypervariable regions separated by an intermediate conserved region [16]. ORF3 (7217–7634 nucleotides) encodes another capsid protein, VP2, which is essential for viral infection of host cells and viral particle assembly.

VP1 is the major capsid protein of FCV, and contains relatively conserved regions (B, D, and F) as well as highly variable regions (C and E). Region E contains a 5′ hypervariable region (HVR), a central conserved region and a 3′ HVR. It has been suggested that strains causing upper respiratory tract disease (URTD) and virulent systemic disease (VSD) differ in the physicochemical properties of seven amino acids in region E [17], which are associated with receptor binding and cell entry [18]. Region E contains linear B-cell epitopes [19], and mutations in these regions can result in immune evasion, partly explaining the limited efficacy of vaccines. Region E is exposed on the VP1 protein surface, contributes to most of the antigenic variability and is essential for binding to the FCV receptor, feline junctional adhesion molecule-A (fJAM-A) [20]. Therefore, a comprehensive understanding of the genetic diversity and evolutionary characteristics of region E is crucial for vaccine development, antibody production, immunodiagnostic testing and the study of FCV pathogenesis.

This study investigates the epidemiological status and molecular characteristics of FCV in Beijing, which can provide a theoretical basis for understanding the prevalence of FCV and formulating further prevention and control measures.

## 2. Materials and Methods

### 2.1. Sample Information and Treatment

This study collected diagnostic results and clinical information from 402 cats. Cats exhibiting URTD symptoms, mouth ulcers or chronic gingivostomatitis were screened for FCV by qRT-PCR at the China Agricultural University Veterinary Teaching Hospital (CAUVTH) between June and December 2023. A sample was considered FCV-positive if the Ct ≤ 36 and a specific amplification curve was observed by the detection kit from Harbin Anheal Biotechnology Co., Ltd. (Harbin, China). Some samples were also tested for feline herpesvirus, Mycoplasma and Chlamydia using the detection kit from Harbin Anheal Biotechnology Co., Ltd. The data included age, gender, breed, number of cats in the household, vaccination status and clinical symptoms. Nasal and oropharyngeal swabs from FCV-positive cats were stored at −80 °C for virus isolation.

### 2.2. Statistical Analysis

Statistical analyses were performed using R software (version 2023.06.2). Chi-square tests were used to analyze the associations between FCV infection and variables such as age, gender, vaccination status, number of cats in the household and breed. Variables with *p* < 0.30 were considered for inclusion in the multivariable analysis [21]. The multivariable analysis was conducted using a logistic regression model. The model’s goodness-of-fit was assessed using the Hosmer–Lemeshow test, with *p* > 0.05 indicating an adequate model fit. Odds ratios (ORs) and 95% confidence intervals (CIs) were calculated and *p* < 0.05 was considered statistically significant.

### 2.3. Virus Isolation

We attempted to isolate FCV from all the positive swabs screened by qRT-PCR. After thoroughly twirling the FCV-positive swab samples, the swabs were discarded and the samples were centrifuged at 8000× *g* for 10 min at 4 °C. The supernatant was then filtered through a 0.22 μM membrane and inoculated onto a monolayer of Crandell Reese feline kidney (CRFK) cells cultured in DMEM supplemented with 10% fetal bovine serum (i-presci, Beijing, China), penicillin (100 U/mL) and streptomycin (0.1 mg/mL) at 37 °C with 5% CO_2_ incubation. Cytopathic effects (CPEs) were monitored daily. If no CPEs were observed after three consecutive passages, the sample was deemed negative. For cultures exhibiting CPEs, the samples underwent three rounds of freeze–thaw cycles, followed by centrifugation at 4000× *g* for 10 min. The supernatant was collected and stored at −80 °C for further experiments

### 2.4. Genome Amplification and Sequencing

Based on the FCV sequences from NCBI, three pairs of primers covering the entire sequence were designed using SnapGene software (version 6.0.2), with reference to previously published literature [22]. The primers were synthesized by Shanghai Sangon Biotech (Shanghai, China), and the sequences are listed in Table 1. The three amplified fragments were named P1, P2 and P3, respectively.

A total of 200 μL of cell culture supernatant was collected for RNA extraction. Total RNA was extracted using the Virus Genome DNA/RNA Extraction Kit (Aidlab, Beijing, China) according to the manufacturer’s instructions. The RNA was reverse transcribed into cDNA using L-MLV Reverse Transcriptase (Promega, Madison, WI, USA).

The reverse-transcribed cDNA was amplified using 2× Taq Master Mix (Vazyme, Nanjing, China) to generate the three overlapping fragments. The total PCR reaction volume was 25 μL, consisting of 12.5 μL of 2× Taq Master Mix, 1 μL of 10 μM forward primer, 1 μL of 10 μM reverse primer, 1.5 μL of template cDNA and 9 μL of double-distilled water. The PCR program included an initial denaturation at 94 °C for 5 min, followed by 35 cycles of denaturation at 95 °C for 30 s, annealing at 55 °C for 30 s (with the P2 fragment annealing at 57 °C) and extension at 72 °C for 180 s, with a final extension at 72 °C for 5 min. The PCR products were verified by 1% agarose gel electrophoresis to confirm the target band size. The PCR products generating the target band were sequenced using the Sanger sequencing approach (Sangon Biotech, Shanghai, China).

### 2.5. FCV Sequences Analysis

The three sequencing results were assembled into a full-length FCV sequence and nucleotide and amino acid sequence alignments were performed by the MegAlign program in Lasergene software 7.2 (DNASTAR, Madison, WI, USA). A phylogenetic analysis of full-length FCV genomic sequences was performed using MEGA11.0 software (Tempe, AZ, USA) with the distance-based neighbor-joining method. The bootstrap values were calculated on 1000 replicates. All 100 reference sequences were downloaded from GenBank for different countries and regions over the past 30 years. These reference sequences included 64 domestic Chinese isolates, 33 foreign isolates, and 3 vaccine strains, with 10 strains identified as VSD isolates. The resulting phylogenetic tree was visualized and modified using the online website (iTOL: Interactive Tree Of Life, https://itol.embl.de/, accessed on 9 March 2024). To identify potential recombination events, RDP4 (version 4.1, Martin DP, Cape Town 7549, South Africa) and Simplot software (version 3.5.1, JHK University, Baltimore, MD, USA) were used to compare the full-genome sequences of FCV isolates with the reference strains with a 200 bp window and 20 bp steps. Phylogenetic trees based on each recombinant fragment were constructed to confirm the accuracy of the recombination events.

## 3. Results

### 3.1. Epidemiological Investigation

Among the 402 clinical samples, 126 were positive for FCV (31.3%). Of these, 76 cases were solely infected with FCV and exhibited symptoms such as sneezing, coughing, tearing, nasal discharge, oral ulcers, anorexia and fever. Mixed infections with other respiratory pathogens were identified in 50 cases. Specifically, five cats were co-infected with feline herpesvirus, thirty-two with Mycoplasma, and four with Chlamydia. Additionally, six cats were co-infected with both feline herpesvirus and Mycoplasma, one with both feline herpesvirus and Chlamydia, and one with both Mycoplasma and Chlamydia. One case was simultaneously infected with all four pathogens.

Univariate analysis using chi-square tests showed that vaccination status, gender, number of cats in the household, breed and age were associated with FCV infection (Table 2). These variables were subsequently included in the logistic regression model, and the results are presented in Table 3. The logistic regression analysis revealed that cats with improper vaccination or no vaccination had a 2.79-fold (*p* = 0.001) and 2.97-fold (*p* = 0.002) higher risk of FCV infection, respectively, compared to cats that were properly vaccinated. Cats in households with two cats or more than two cats had a 1.85-fold (*p* = 0.021) and 3.47-fold (*p* < 0.001) higher risk of FCV infection, respectively, compared to single-cat households. Cats aged 4 to 12 months had a 2.20-fold (*p* = 0.007) higher risk of FCV infection compared to those older than one year. In this study, gender and breed were not statistically associated with FCV infection.

### 3.2. Virus Isolation, Amplification and Sequencing

**Figure 1:**
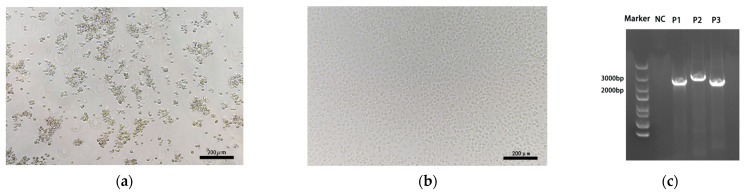
Isolation and amplification of FCV in this study. CRFK cells were inoculated with FCV (**a**) and uninfected medium (**b**) at 24 h postinfection. (**c**) FCV isolate was amplificated by RT-PCR with 3 fragments. NC indicates the negative control.

CRFK cells infected with FCV strains exhibited CPE within 1–2 days, characterized by cell rounding, aggregation, and the typical “grape-like” clustering pattern. Eventually, the cells detached from the culture dish surface and floated in the culture medium (Figure 1a). No CPEs were observed in the control group (Figure 1b). The sizes of the three PCR products used for full-length sequencing matched the expected values, as shown in Figure 1c. The sequencing results were assembled to generate 60 full-length FCV genomes, which have been uploaded to the GenBank database. The corresponding accession numbers are PP554894–PP554953.

### 3.3. Phylogenetic and Homology Analysis of FCV Isolates

**Figure 2:**
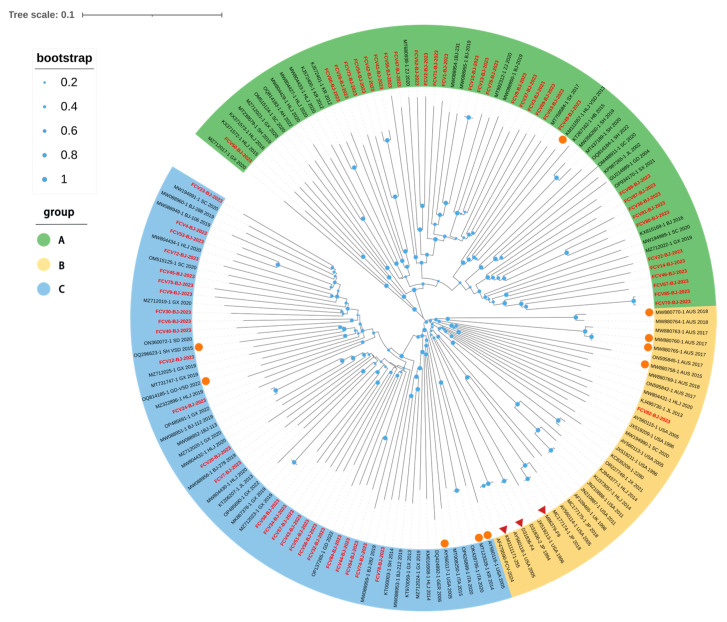
Phylogenetic tree based on full-length genomic sequence of 60 isolates and 100 FCV reference strains available in GenBank via the NJ method using the Kimura two-parameter model in the MEGA 11.0 software (Tempe, AZ, USA) package with 1000 bootstraps. The isolates in this study are shown in “red” and the vaccine strains are labeled with a “red triangle” and the VSD strains are labeled with an “orange circle”. The scale bar indicates the number of nucleotide substitutions per site. Bootstrap values are represented by blue circles of different diameters at each clade node.

A full-genome phylogenetic tree was constructed for the 60 isolates and 100 reference strains (Figure 2). Based on genetic relationships, the FCV isolates were categorized into three groups. Among the 60 isolates, 33 strains were classified as Group A, 1 strain as Group B, and 26 strains as Group C. Group A includes isolates exclusively from China and South Korea. Specifically, fcv50 and the Guangxi isolate (MZ712017) clustered together, while fourteen isolates in this study formed a distinct branch with three isolates from Beijing (2019) and two isolates from Zhejiang Province, China (2020). The fcv-26/41/42/54/66/73 isolates were grouped with South Korean isolates in the same branch, which also contained isolates from Heilongjiang Province, China (2020), showing a high homology among these isolates. The fcv49 isolate and the 2017 Shaanxi isolate (MT759584) were placed in the same branch, which also included isolates from Shanghai, Hubei and Heilongjiang provinces in China. The fcv36/80/81/86/87 isolates and the 2021 Shaanxi isolate (OP934170) were grouped into the same branch, while the fcv14/22/46/67/70/85 isolates clustered with isolates from Sichuan and Guangxi province. Group C included isolates from China, as well as some from Europe, the United States and South Korea. The 26 isolates from this study were all placed in different branches from foreign isolates, but they clustered with other domestic isolates from various regions of China. Notably, only one isolate from this study clustered with the widely used domestic vaccine strain FCV-255 and was placed in a separate branch within Group B. The remaining 59 isolates were distributed across the other two groups. These results suggest that the isolates in this study exhibit a radial distribution and are genetically distant from the vaccine strain.

To investigate the genetic variation in FCV strains in the Beijing region, nucleotide homology comparisons were conducted on the 60 full-length sequences using MegAlign software (version 7.1.0). The nucleotide identity among these sequences ranged from 75.7% to 99.9%. Specifically, the nucleotide and amino acid identities of the ORF1 fragment among the 60 isolates ranged from 76.1% to 99.9% and 87.6% to 100%, respectively; for ORF2, they ranged from 72.4% to 100% and 79.5% to 99.9%, respectively; and for ORF3, from 70.1% to 100% and 75.2% to 100%, respectively. When compared with 100 reference strains from the GenBank database, the nucleotide identity ranged from 75.4% to 88.5%. Specifically, the nucleotide identities with the vaccine strains FCV255, F9, and F4 ranged from 76.2% to 79.7%, 75.7% to 79.7%, and 76.5% to 79.8%, respectively. The nucleotide identity with 10 strains assumed to be VSD ranged from 75.9% to 87.3%.

**Figure 3:**
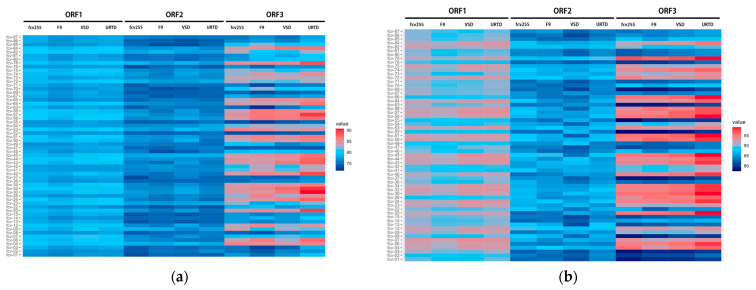
Heatmap based on the nucleotide sequence (**a**) and amino acid (**b**) identities of each isolation with 4 represent FCV strains. The bar on the right represents sequence identity (blue for lower identities and red for higher identities) among different strains.

To further explore the genomic characteristics of the Beijing isolates, nucleotide and amino acid homology analyses of FCV ORFs were conducted on the 60 to compare them with the widely used domestic vaccine strain FCV-255, the vaccine strain F9, a representative VSD strain (AY560117.1) and a representative URTD strain (AY560113.1) (Figure 3). The results showed that the nucleotide homology of the ORF2 fragment between the 60 isolates and the reference strains ranged from 73.5% to 78.9% for FCV-255, 73.4% to 79% for F9, 73.5% to 79.2% for the VSD strain and 73.8% to 78.4% for the URTD strain. The amino acid homology ranged from 82.2% to 89.4%, 82.6% to 89.2%, 80.8% to 88.2% and 83.1% to 89.4%, respectively. Heatmap analysis of the homology results revealed that in the three ORFs, the 60 isolates showed lower nucleotide and amino acid homology with the reference strains in ORF2, which encodes the major capsid protein. In contrast, ORF3, which encodes the minor capsid protein, showed a higher homology, while ORF1, encoding non-structural proteins, exhibited an intermediate homology. These findings suggest that the ORF2 region exhibits substantial variability, which may be associated with the low efficacy of vaccines observed previously.

### 3.4. Amino Acid Analysis of the VP1 Protein

**Figure 4:**
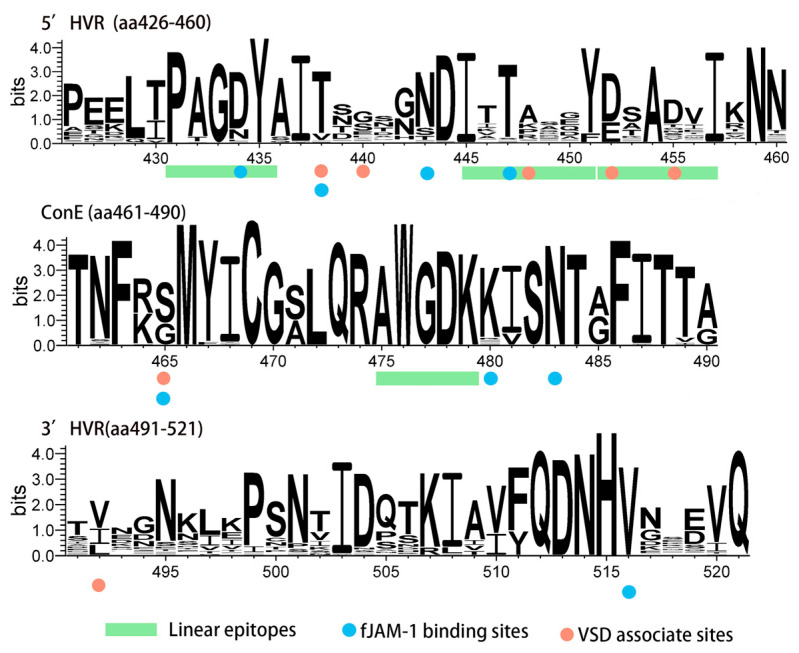
Amino acid sequence conservation of region E. The sequence logo was generated from the alignment of 60 full–length VP1 strains. Green bands indicate linear epitopes. Blue circles indicate fJAM-A binding sites. Red circles indicate VSD–associated sites. Logo plots were generated using WebLogo 3.

The amino acid sequence of the VP1 protein from the isolates was compared with the widely used domestic vaccine strain FCV-255. Among the isolates, twenty-five strains had a Gly insertion at position 127aa in the B region, while seven isolates had an Asn or Val insertion at positions 503aa or 504aa in the E region. Linear epitope analysis revealed that the amino acid sequences of the epitopes 426aa–435aa and 475aa–479aa were highly conserved, while there were significant differences in the sequence of the 445aa–457aa region containing both neutralizing antibody epitopes (445aa–451aa, ITTANQY) which can be recognized by neutralizing monoclonal antibodies [16] and the binding site for the fJAM-A receptor. Additionally, this region includes three amino acid sites that are potentially associated with VSD (Figure 4).

The genetic background differences between FCV-VSD and FCV-URTD have not been conclusively determined. However, studies have shown that FCV-VSD and FCV-URTD exhibit physicochemical differences at seven amino acid positions in the E region [17], which affect antigenic variation and the binding process to the fJAM-A receptor. In this study, 14 strains exhibited more than three amino acid variations that were consistent with the VSD type described in previous research (Table A1). However, these infected cats did not show clinical symptoms typical of VSD. The findings of this study suggest that there is no direct correlation between the clinical symptoms of FCV and its genetic background.

### 3.5. Detection of Recombinant of FCV Isolates

**Figure 5:**
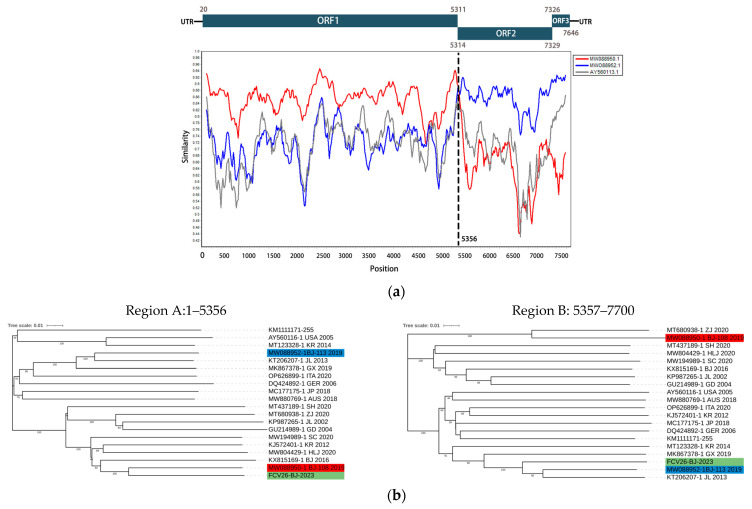
Recombination analysis of FCV-26 identified in this study. (**a**) Genome scale similarity comparisons of FCV-26 with FCV-BJ-108 isolate (red) and FCV-BJ-113 isolate (blue) using a sliding window. AY560113.1 is shown as a gray line as a reference strain. (window size: 200 bp, step size: 20 bp). Potential recombination breakpoints were marked by a black vertical dotted line with nucleotide sites at the bottom. (**b**) ML phylogenetic trees based on every recombinant fragment within FCV-26 and 17 reference FCV isolates are shown below the similarity plot. The isolate FCV-26 is labeled with a green background, and the putative recombinant major parent isolate FCV-BJ-108 is marked with a red background. The putative recombinant minor parent isolate FCV-BJ-113 is marked with a blue background.

RDP4 (version 4.1) and SimPlot software (version 3.5.1) were used to detect potential recombination events. The results indicated that FCV-26 is a potential recombinant virus, originating from recombination between MW088950.1 and MW088952.1, with *p*-values from all seven detection methods being less than 1 × 10^−10^ (Table 4). The recombination site between MW088950.1 and MW088952.1 is located at nucleotide position 5356, at the junction of ORF1 and ORF2, consistent with commonly observed recombination sites in caliciviruses, as reported in previous studies [23,24]. This potential recombination site divides FCV-26 into two regions: Region A (nucleotides 1–5356), closely related to the MW088950.1 strain, and Region B (nucleotides 5357–7690), closely related to the MW088952.1 strain. Notably, both parent strains, MW088950.1 and MW088952.1, were isolated in Beijing in 2019, representing the first documented recombination event in this region and suggesting the possibility of recombination and cyclic infection among FCV strains in Beijing. The recombination event was further confirmed using SimPlot software, and phylogenetic trees for Region A and B were constructed using the maximum likelihood method (Figure 5).

## 4. Discussion

The FCV prevalence in this study was 31.3%, higher than the 26.0% prevalence in Jiangsu, China [25], and 25.7% in Moscow [26], but lower than the 43.0% prevalence in Hangzhou, China [27], and 45.0% in Switzerland [28]. Compared to the 44.3% prevalence in Beijing in 2019 [29], the rate has decreased, possibly due to increased vaccination awareness and improved management among pet owners in Beijing. The high prevalence of FCV is linked to its high genetic variability [30], persistent viral shedding during the recovery period in infected cats [31] and reinfection with either variant strains of the same virus or different viral strains [32]. Prevalence rates in different regions may vary due to factors such as geographic location, sampling time, and sample size.

We analyzed the risk factors for FCV infection, and the results showed that proper vaccination effectively prevents FCV infection, consistent with previous studies [25,27]. The high infectivity and genetic variability of FCV make multi-cat households more vulnerable to infection. Multivariate logistic regression analysis indicated that the risk of FCV infection in households with two cats and those with three or more cats was 1.85 and 3.47 times higher, respectively, compared to single-cat households. This emphasizes the importance of environmental management and disease monitoring, particularly in multi-cat households. Previous studies have shown that cats under one year of age are more vulnerable to FCV infection [25,32]. However, this age classification may overlook the protective effect of maternally derived antibodies. Considering the decline in maternal antibody levels [33], we divided the under-one-year age group into two subcategories: 0–3 months and 4–12 months. We found that cats in the 4–12 months age group were more likely to be infected with FCV. This may be due to the loss of maternal antibodies in cats aged 4–12 months [33], coupled with the lack of timely vaccination, leading to an inability to develop effective humoral immunity. Cats aged 4–12 months are more likely to leave catteries for trade or other transport activities, increasing their risk of exposure to pathogens. Studies have shown that maternally derived antibodies can reduce the efficacy of FCV vaccines [34], making it recommended to vaccinate kittens after 8 weeks of age for optimal immune protection. We also found that cats with oral symptoms were more likely to be infected with FCV. This may be due to FCV binding to fJAM-A receptors at tight junctions between endothelial and epithelial cells, disrupting cell integrity and intercellular connections [35], which leads to symptoms such as oral ulcers, tongue ulcers, and gingivitis

Full genome phylogenetic analysis showed that the 60 isolates in this study were distributed across three groups, with isolates from this study present in all three. However, the C group, which includes the widely used vaccine strain FCV-255, contained only one isolate, which was placed on a different branch. This suggests that the FCV strains circulating in Beijing exhibit significant genetic divergence from the vaccine strain. Previous studies have shown that greater antigenic differences between field strains and vaccine strains correlate with lower cross-protection provided by the vaccine [36,37]. The fact that some immunized cats remain infected with FCV indicates that current vaccines may not provide sufficient protection. There have been varying opinions on the cross-protection of vaccines in previous studies [38,39,40], and further in vitro virus neutralization tests are needed to verify these findings. Consistent with many previous studies [4,25,27], this research found no clear correlation between the phylogenetic tree clustering and geographic location. Notably, six isolates in this study clustered with two isolates from the South Korea in a branch of Group A, which also included isolates from Northeast China. Given the proximity of these regions, we speculate that this FCV branch is widely distributed in both Northern China and South Korea. As global exchanges increase and become more frequent, the geographic isolation of FCV strains is becoming less distinct. This blurring of geographic boundaries increases the risk of FCV recombination and variation, posing new challenges for FCV infection prevention.

The analysis of amino acid site conservation in the E region revealed that sites associated with fJAM-A binding are relatively conserved, which may represent an evolutionary strategy enabling FCV to efficiently bind to and infect host cells. The E region contains four linear epitopes involved in immune recognition, with amino acids at positions 431–435 (PAGDY) being highly conserved non-neutralizing epitopes, and amino acids at positions 445–451 (ITTANQY) being highly variable neutralizing epitopes [41]. This is consistent with our analysis of amino acid site conservation in the E region. Notably, 16 strains exhibit a pair of aa mutations (Ala-448-Lys/Arg and Asp-452-Glu). These mutations result in electrostatic attraction between the positively charged Lys/Arg and the negatively charged Glu, potentially affecting the stability of the α-helix, which warrants further investigation. Overall, the high variability of neutralizing epitopes reduces the cross-protection offered by vaccines, potentially leading to vaccine failure. While non-neutralizing epitopes are located in highly variable regions, they are highly conserved. Previous studies have shown that these epitopes have broad cross-reactivity with antibodies [16], suggesting their significant potential for diagnosing FCV infections.

No clinical signs of VSD were observed in any of the infected cats in this study. However, several isolates exhibited amino acid mutations at multiple sites consistent with seven aa sites assumed to be associated with VSD strains [17]. No mutations could clearly distinguish URTD from VSD phenotypes in three FCV-VSD outbreaks in Australia [10], which suggests that there is no clear association between VSD and the seven amino acids identified. The genetic relationship between URTD and VSD needs to be explored with more data and the established reverse genetics for FCV may help verify the genetic distinctions between these two forms of disease. Recombination is a widespread phenomenon among RNA viruses and plays a crucial role in their variation and evolution [42]. For the first time, we identified a recombination event in FCV isolates from the Beijing region. The recombination site was located at the junction of ORF1 and ORF2, consistent with common recombination hotspots observed in the *Caliciviridae* family [43]. Both parental strains were isolated from Beijing in 2019, suggesting the presence of multiple co-circulating FCV strains and the occurrence of inter-strain recombination. As an important model virus within the *Caliciviridae* family, FCV merits further research to better understand its recombination mechanisms. Such studies are crucial for advancing our knowledge of other *Caliciviridae* family pathogens, including Norovirus.

## 5. Conclusions

In summary, this study investigates the epidemiological characteristics of FCV in Beijing, China. We conduct a full-genome phylogenetic analysis and amino acid characterization of the major capsid protein in 60 FCV isolates. The results demonstrate genetic diversity among FCV strains in Beijing and, for the first time, we identify a recombination event in FCV isolates from this region. These findings provide valuable insights that may guide the development of more effective prevention and control strategies for FCV.

## Figures and Tables

**Table 1:** Primers for sequencing the complete genome of FCV.

Primer	Sequence (5′-3′)	Position	Amplicon Size (bp)
P1-F	GTAAAAGAAATTTGAGACAATGTCTCAAAC	1–30	2556
P1-R	TTGTCAGGGGCAGTAAGCACATC	2534–2556	
P2-F	GTGGTAACCGTTAATTCGGTGTTT	2441–2464	2897
P2-R	CACGTTAGCGCAGGTTGAGCAC	5316–5337	
P3-F	CAACAGCCAGTTTAATGGTGTG	5221–5242	2463
P3-R	CCCTGGGGTTAGGCGC	7668–7683	

**Table 2:** Univariate analysis of factors associated with feline calicivirus infection.

Variable	Category	Proportion	Frequency (%)	χ2	*p* Value
Overall		126/402	31.3		
Age	0–3 months	32/109	29.4	3.3932	0.1833
	4–12 months	49/131	37.4		
	>1 years	45/162	27.8		
Gender	Male	83/247	33.6	1.2602	0.2616
	Female	43/155	27.7		
Vaccination	Unvaccinated	55/148	37.3	11.581	0.003056
	Not proper	47/131	35.9		
	Proper	24/123	18.2		
Residential density	≥3 cats	30/58	51.7	16.395	0.0002754
	2 cats	38/110	34.5		
	1 cat	58/234	24.8		
Breed	Chinese domestic cats	53/136	39.0	12.18	0.0324
	British shorthair	23/94	24.5		
	Ragdoll	15/40	37.5		
	American shorthair	4/33	12.1		
	Doven	7/24	29.2		
	Others	24/75	32.0		

Variables with *p* < 0.30 were considered for inclusion in the multivariable analysis.

**Table 3:** Multivariate logistic regression analysis of factors associated with feline calicivirus infection.

Variable	OR	95%CI	*p* Value
Age			
0–3 months	0.99	0.51–1.94	0.985
4–12 months	2.20	1.25–3.94	0.007
>1 years	1.0 *		
Gender			
Male	1.41	0.88–2.28	0.160
Female	1.0 *		
Vaccination			
Unvaccinated	2.97	1.49–6.03	0.002
Not proper	2.79	1.52–5.29	0.001
Proper	1.0 *		
Residential density			
≥3 cats	3.47	1.80–6.76	<0.001
2 cats	1.85	1.09–3.13	0.021
1 cat	1.0 *		
Breed			
Chinese domestic cats	1.16	0.60–2.28	0.664
British shorthair	0.85	0.41–1.75	0.662
Ragdoll	1.52	0.64–3.60	0.339
American shorthair	0.33	0.08–1.03	0.074
Doven Rex	0.98	0.33–2.75	0.972
Others	1.0 *		

*p* < 0.05 was considered significant. OR, odds ratio; CI, confidence interval. * Reference category.

**Table 4:** Information on recombination events of FCV-26 detected by RPD4 software.

Recombinant	Major (Similarity)	Minor (Similarity)	*p*-Value of 7 Detection Methods						
			RDP	GENECONV	BootScan	MaxChi	Chimaera	SiScan	Phylpro
FCV-26	MW088950.1	MW088952.1	1.661 × 10^−54^	1.152 × 10^−23^	2.789 × 10^−48^	3.192 × 10^−25^	4.049 × 10^−29^	8.496 × 10^−33^	1.248 × 10^−10^

## Data Availability

All data and results related to this study are included in the article. Raw data are available from the corresponding author upon reasonable request.

## Appendix A

**Table A1:** Comparison of amino acids in region E of FCV capsid proteins with VSD strains.

Isolate	Amino-Acid Residues and Physico-Chemical Properties Associated with VSD Pathotype						
	438	440	448	452	455	465	492
VSD-FCV	V	Q	K/R	E	T	S	V
URTD-FCV	T	G	A	D	D	G	V
FCV1-BJ 2023	T	S	G	D	D	S	I
FCV2-BJ 2023	A	S	A	D	G	S	V
FCV3-BJ 2023	V	S	P	D	D	G	V
FCV4-BJ 2023	T	S	R	E	D	G	I
FCV5-BJ 2023	T	S	R	E	D	G	V
FCV6-BJ 2023	T	G	A	D	T	S	V
FCV7-BJ 2023	T	G	A	D	D	S	V
FCV8-BJ 2023	T	G	A	D	D	S	I
FCV9-BJ 2023	T	S	A	D	D	G	V
FCV12-BJ2023	T	G	A	D	S	S	V
FCV13-BJ2023	T	S	A	D	S	S	V
FCV14-BJ2023	T	E	K	E	T	S	L
FCV15-BJ2023	T	S	A	D	S	S	V
FCV20-BJ 2023	T	G	S	D	D	S	V
FCV22-BJ 2023	T	Q	A	E	T	S	L
FCV23-BJ 2023	T	S	K	E	D	G	I
FCV24-BJ 2023	T	E	P	D	N	S	V
FCV26-BJ 2023	T	G	A	D	D	S	I
FCV30-BJ 2023	T	E	P	D	V	S	V
FCV32-BJ 2023	T	E	A	D	T	S	V
FCV34-BJ 2023	T	G	A	D	D	S	V
FCV36-BJ 2023	T	G	A	D	D	G	L
FCV37-BJ 2023	T	E	P	D	V	S	I
FCV40-BJ 2023	T	G	A	D	D	S	V
FCV41-BJ 2023	T	G	K	E	D	S	I
FCV42-BJ 2023	T	G	K	E	D	S	I
FCV43-BJ 2023	T	T	A	D	D	S	V
FCV45-BJ 2023	T	S	R	E	D	G	I
FCV46-BJ 2023	V	Q	K	E	I	S	L
FCV47-BJ 2023	T	G	A	D	D	G	V
FCV49-BJ 2023	T	S	S	D	D	G	L
FCV50-BJ 2023	T	G	A	D	D	S	V
FCV51-BJ 2023	T	G	A	D	D	S	V
FCV52-BJ 2023	T	N	A	D	D	S	V
FCV53-BJ 2023	T	S	R	E	D	G	I
FCV54-BJ 2023	T	L	R	E	N	S	I
FCV55-BJ 2023	T	A	P	D	D	G	I
FCV56-BJ 2023	T	G	P	E	D	S	V
FCV57-BJ 2023	V	E	A	D	V	S	I
FCV58-BJ 2023	T	G	S	D	S	S	V
FCV63-BJ 2023	T	A	A	D	D	S	V
FCV64-BJ 2023	T	S	P	D	D	S	V
FCV66-BJ 2023	T	S	L	E	D	G	V
FCV67-BJ 2023	T	Q	K	D	L	S	L
FCV69-BJ 2023	T	G	P	D	D	S	V
FCV70-BJ 2023	V	L	K	E	Q	S	L
FCV71-BJ 2023	T	G	A	D	D	G	I
FCV72-BJ 2023	T	S	K	E	E	G	I
FCV73-BJ 2023	V	E	R	E	N	S	V
FCV74-BJ 2023	T	E	A	D	G	S	V
FCV75-BJ 2023	T	L	R	E	D	G	I
FCV76-BJ 2023	L	L	A	D	T	S	I
FCV78-BJ 2023	T	G	R	E	D	S	V
FCV80-BJ 2023	T	K	A	D	D	G	L
FCV81-BJ 2023	T	S	A	D	D	G	L
FCV82-BJ 2023	T	A	A	D	G	G	I
FCV84-BJ 2023	T	G	A	D	N	S	V
FCV85-BJ 2023	V	L	K	E	Q	S	L
FCV86-BJ 2023	T	G	P	D	Q	G	L
FCV87-BJ 2023	T	G	A	D	G	G	L

Mutations consistent with VSD are highlighted with a gray background.
